# CHESS: a new human gene catalog curated from thousands of large-scale RNA sequencing experiments reveals extensive transcriptional noise

**DOI:** 10.1186/s13059-018-1590-2

**Published:** 2018-11-28

**Authors:** Mihaela Pertea, Alaina Shumate, Geo Pertea, Ales Varabyou, Florian P. Breitwieser, Yu-Chi Chang, Anil K. Madugundu, Akhilesh Pandey, Steven L. Salzberg

**Affiliations:** 1 Center for Computational Biology, McKusick-Nathans Institute of Genetic Medicine, Johns Hopkins University School of Medicine, Baltimore, MD USA; 2 Department of Biomedical Engineering, Johns Hopkins University, Baltimore, MD USA; 3 Department of Computer Science, Johns Hopkins University, Baltimore, MD USA; 4 McKusick-Nathans Institute of Genetic Medicine, Johns Hopkins University School of Medicine, Baltimore, MD USA; 5 Institute of Bioinformatics, International Technology Park, Bangalore, India; 6 Manipal Academy of Higher Education (MAHE), Manipal, Karnataka India; 7 Departments of Biological Chemistry, Pathology, Neurology, and Oncology, Johns Hopkins University School of Medicine, Baltimore, MD USA; 8 Present address: Center for Individualized Medicine and Department of Laboratory Medicine and Pathology, Mayo Clinic, Rochester, MN USA; 9 Department of Biostatistics, Bloomberg School of Public Health, Johns Hopkins University, Baltimore, MD USA

**Keywords:** Human gene count, GTEx, RNA sequencing, Transcriptome, Transcriptome assembly

## Abstract

We assembled the sequences from deep RNA sequencing experiments by the Genotype-Tissue Expression (GTEx) project, to create a new catalog of human genes and transcripts, called CHESS. The new database contains 42,611 genes, of which 20,352 are potentially protein-coding and 22,259 are noncoding, and a total of 323,258 transcripts. These include 224 novel protein-coding genes and 116,156 novel transcripts. We detected over 30 million additional transcripts at more than 650,000 genomic loci, nearly all of which are likely nonfunctional, revealing a heretofore unappreciated amount of transcriptional noise in human cells. The CHESS database is available at http://ccb.jhu.edu/chess.

**Electronic supplementary material:**

The online version of this article (10.1186/s13059-018-1590-2) contains supplementary material, which is available to authorized users.

## Background

Scientists have been attempting to estimate the number of human genes for more than 50 years, dating back to 1964 [1]. In the decade preceding the initial publication of the human genome, multiple estimates were made based on sequencing of short messenger RNA fragments, and most of these estimates fell in the range of 50,000–100,000 genes [2–5]. When the human genome was published in 2001, the estimates of the gene count were dramatically lower, with one paper reporting 31,000 genes [6] and the other 26,588 plus ~ 12,000 genes with “weak supporting evidence” [7]. As the genome was gradually made more complete and the annotation improved, the number continued to fall; when the first major genome update was published in 2004, the estimated gene count was revised to 24,000 [8]. Later efforts suggested that the true number of protein-coding genes was even smaller: a 2007 comparative genomics analysis suggested 20,500 [9], and a proteomics-based study in 2014 estimated 19,000 [10].

One striking feature of most early attempts to catalog all human genes was their lack of precision. Most estimates have only one to two significant digits, indicating major uncertainty about the exact number. As we reported in 2010, the estimates of the human gene count at that time averaged ~ 22,500 genes [11]. As of late 2017, one of the most reliable catalogs of human genes, the curated reference set from NCBI’s RefSeq database [12], contained 20,054 distinct protein-coding genes, and another widely used human gene catalog, GENCODE [13], contained 19,817. The international CCDS database, an ongoing effort to identify all human and mouse genes [14], listed 18,894 human protein-coding genes in March 2018 (release 20).

The human gene list has a tremendous impact on biomedical research. A huge and still growing number of genetic studies depend on this list, for example:

- Exome sequencing projects use exon capture kits that target most “known” exons. Any exons that are not listed in standard human annotation are ignored.
- Genome-wide association studies (GWAS) attempt to link genetic variants to nearby genes, relying on standard catalogs of human genes.
- Many software packages that analyze RNA sequencing (RNA-seq) experiments, which measure gene expression, rely on a database of known genes and cannot measure genes or splice variants unless they are included in the database.
- Efforts to identify cancer-causing mutations usually focus on mutations that involve known genes, ignoring mutations that occur in other regions.

These and other examples encompass thousands of experiments and an enormous investment of time and effort. The creation of a more complete, accurate human gene catalog will have an impact on many of these studies. For example, exome sequencing studies targeting Mendelian diseases, which should be the easiest diseases to solve, have reported diagnostic success in only about 25% of cases [15, 16], perhaps because many exons and genes are excluded from exome capture kits. A better gene list may also help to explain the genetic causes of the many complex diseases that have thus far remained largely unexplained, despite hundreds of large GWAS and other experiments.

As part of the creation of a human gene list, we must first define what is meant by the term “gene.” During the Human Genome Project, most efforts to estimate and annotate genes focused on protein-coding genes, i.e., regions of the genome that are transcribed into RNA and then translated into proteins. At the time, most scientists assumed that non-coding genes represented only a very small portion of the functional elements of the human genome and that most RNA genes (e.g., transfer RNAs and ribosomal RNA genes) were already known. A few years after the initial publication of the human genome, though, scientists began to uncover a large and previously unappreciated complement of long noncoding RNA genes, lncRNAs [17, 18], which quickly grew to include thousands of novel genes. These genes have a wide range of functions that are just as vital to human biology as many protein-coding genes [19], and any comprehensive list of human genes should include them.

Thus, for the purposes of our study, genes will include any interval along the chromosomal DNA that is transcribed and then translated into a functional protein or that is transcribed into a functional RNA molecule. By “functional,” we mean to include any gene that appears to perform a biological function, even one that might not be essential. We recognize that the proper determination of function can be a lengthy, complex process and that at present, the function of many human genes is unknown or only partially understood. Our definition intentionally excludes pseudogenes, which are gene-like sequences that may arise through DNA duplication events or through reverse transcription of processed mRNA transcripts. Following previous conventions [11], when multiple proteins or RNA genes are produced from the same region through alternative splicing or alternative transcription initiation, we will count these variants as part of a single gene. Our total gene count, therefore, corresponds to the total number of distinct chromosomal intervals, or loci, that encode either proteins or noncoding RNAs; in addition, we report the total number of gene variants, which includes all alternative transcripts expressed at each locus. (In the few cases where distinct genes occupy overlapping intervals, we count these as separate genes.)

## Construction and contents of CHESS

The basis for our human gene catalog is a new analysis of a large, comprehensive survey of gene expression in human tissues, the genotype-tissue expression (GTEx) study, which included samples from dozens of tissues collected from hundreds of individuals [20]. All of these samples were subjected to deep RNA-sequencing, with tens of millions of sequences (“reads”) captured from each sample, in an effort to measure gene expression levels across a broad range of human cell types. This exceptionally large set of transcript data—just under 900 billion reads—provided an opportunity to construct a new set of human genes and transcripts. We accomplished this by assembling all of the samples, merging the results, and applying a series of computational filters to remove transcripts with insufficient evidence.

During the Human Genome Project, the gold standard for identifying a gene was evidence that it was transcribed into messenger RNA. This was the basis for the first large-scale effort to capture and catalog human genes [21] and for many subsequent efforts. However, over time, it has become clear that the mere fact that a region of the genome is transcribed is insufficient to prove that it has a function, especially in light of evidence that random mutations can easily create transcriptional start sites [22]. A second, arguably more powerful piece of evidence that a sequence is a gene is evolutionary conservation: if a protein sequence has been conserved in other species, this provides strong evidence that the sequence provides a useful function, i.e., that it is a gene. A third line of evidence is reproducibility: if we observe a transcript in multiple samples from multiple individuals, then it is less likely be the result of random transcription. We used each of these lines of evidence in constructing the new gene catalog.

### Novel genes and transcripts

We assembled all 9795 RNA-seq samples from the GTEx collection (see the “Methods” section) and removed all transcripts that overlapped with known protein-coding genes, noncoding genes, or pseudogenes from RefSeq [12] or GENCODE [13]. This process generated 5,081,171 novel transcripts at 668,018 loci, where “novel” means that the transcripts did not overlap any annotated genes in either the RefSeq or GENCODE databases. We then used a variety of criteria, described below, to eliminate transcripts due to “noise” [23], i.e., transcripts produced by low-level transcriptional activity that appears to have no functional utility. This noise is so ubiquitous that some computational methods for analyzing RNA-seq experiments automatically impose a threshold below which they will not report a transcript, even if reads are present [24, 25]. We also eliminated novel transcripts with no introns, which we assumed to be either noise or pseudogenes unless they had high expression levels and contained a potential protein-coding gene, as detailed below. Out of the 5,081,171 novel transcripts, only 139,289 (2.7%) in 41,979 (6.3%) distinct loci had at least one intron.

### Protein-coding genes

To identify potentially novel protein-coding genes, we eliminated transcripts based on a series of relatively strict criteria designed to remove noise, pseudogenes, and alignment artifacts. For each transcript, we used blastx [26] to search all open reading frames against all mammalian proteins in GenBank and in UniProtKB/Swiss-Prot to determine whether any were conserved in other species or elsewhere in the human genome. We required that any novel protein-coding transcript satisfy at least the following criteria:

- The transcript must contain at least one intron, and it must have expression level TPM > 1 in at least one tissue, or alternatively, it may be a single-exon transcript with expression level at least as high as the outliers for known transcripts, defined as TPM > 13.87 (see Additional file 1).
- The transcript must not be contained in another transcript, unless it is expressed in more samples than all transcripts that contain it.
- The length of the open reading frame (ORF) must be at least 60 amino acids.
- The ORF cannot overlap known LINE or LTR repeat elements or overlap ribosomal RNA genes.
- The BLAST *e*-value of the best protein alignment must be 10^−15^ or smaller.
- If the predicted protein matches another protein, the length of the ORF must be at least 75% of the length of the matching protein (in order to eliminate pseudogenes, which tend to be truncated).
- If predicted transcripts are in conflicting loci (i.e., overlapping transcripts on opposite strands), we only keep those that align to proteins with known functions.

After applying these filters, we were left with 1335 transcripts. Seventy of these transcripts (in 55 genes) contained domains that matched either the Pfam protein families database [27] or the NCBI Conserved Domain Database (CDD) [28] (see Additional files 2 and 3). Upon further screening, we found that the majority of the remaining transcripts overlapped Alu elements [29] or SVA repeat elements [30]. While it is possible that some of these transcripts could encode true protein-coding genes, we chose the conservative approach of eliminating any such transcript if it did not have a Pfam or CDD hit. After this step, the remaining 317 transcripts (which include the 70 that contain Pfam domains) clustered into 224 potentially new protein-coding genes (see Additional files 4 and 5). Combining the 224 new genes with the 20,054 from RefSeq yielded a total of 20,278 potentially protein-coding genes.

**Fig. 1:**
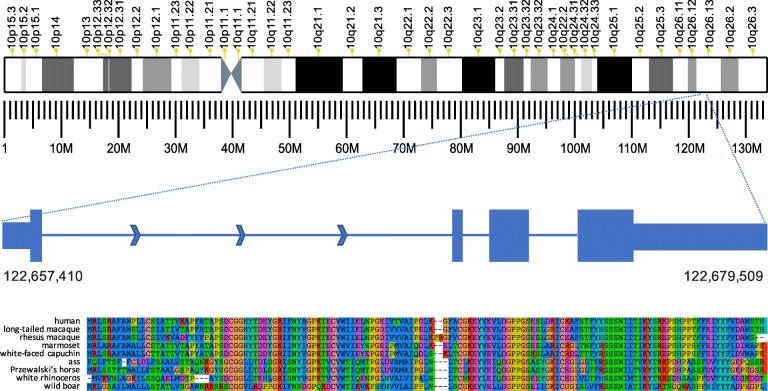
One of 224 new protein-coding genes (CHS.7402) discovered in this study. This 4-exon gene occurs on the forward strand of chromosome 10 at the coordinates shown. The exon lengths are 134, 30, 136, and 663 bp (left to right), with the narrower rectangles indicating the 5′ and 3′ UTR regions. The intron lengths (not shown to scale) are 18,098, 1086, and 1956 bp. The sequence alignment at the bottom shows, top to bottom, the protein sequences from CHS.7402, long-tailed macaque, rhesus macaque, marmoset, white-faced capuchin, ass, Przewalski’s horse, white rhinoceros, and wild boar. The full-length human protein sequence is shown

Figure 1 illustrates one of the novel genes, CHS.7402, discovered by this process. This four-exon gene occurs on chromosome 10 and spans the range 122,657,410–122,679,509, approximately 14 Kb downstream from the nearest known gene, DMBT1. It is highly conserved in multiple other species, with the closest homologs in macaques (94% identical over the full length of the protein, BLAST *e* value 1e−38), followed by marmoset, capuchin, ass, Przewalski’s horse, rhinoceros, wild boar, and others (Fig. 1).

Interestingly, we found no homologous proteins annotated in primates much more closely related to humans such as chimpanzee, gorilla, and orangutan. We searched the transcript sequence of CHS.7402 against the DNA of chimpanzee (*Pan troglodytes*) and found that the sequence matches nearly perfectly, at 97% identity over its entire length, and that chimpanzee also has four exons. Thus, the gene is clearly present, though un-annotated, in *Pan troglodytes*. This illustrates a broader problem with gene annotation: when annotation is created for a new genome, which is typically done through a highly automated process, previously annotated genes from other species provide critical evidence to support the new annotation. Thus, if a gene is missing from the human annotation, it may be omitted from the annotation of other species, especially close human relatives. Multiple sequence alignments for additional novel CHESS proteins are shown in Additional file 1: Figures S6–S8.

We then evaluated the 15,779 lncRNA genes in RefSeq to determine if any of these might instead be protein-coding genes. From all RefSeq lncRNAs, we selected those containing an ORF at least 180 bp (60 amino acids) long and searched these against the mammalian protein database. After excluding read-through transcripts, we found 2762 potential protein sequences that matched a mammalian protein with an *e*-value of 10^−15^ or less and were at least 75% as long as the best matching protein, using the same criteria as used above for novel protein-coding genes.

Because this step was intended only to recover mis-annotated lncRNAs that are likely to be proteins, we retained only those genes for which the best-matching protein had a named function, i.e., we excluded any lncRNA whose best hit was to a protein annotated as hypothetical, unknown, or uncharacterized (see the “Methods” section). After removing hits to proteins that had no associated function, we were able to rescue 53 genes containing 85 transcripts that passed all our criteria for protein-coding regions. Adding these 53 new protein-coding genes to our total, the number of potential protein-coding genes in the human gene catalog increased to 20,331.

Finally, we considered genes from the GENCODE database [13] (releases 25 and 27) that were annotated as “known” protein-coding genes but were missing from RefSeq. Based on proteins that remained in release 27 of GENCODE (see Additional file 6), we found 26 more protein-coding genes, for a total of 20,357. Finally, we subtracted five genes that are present in RefSeq but that appear to be false (discussed below), to yield 20,352 potentially protein-coding genes (Table 1).

**Table 1:** The number of human genes and transcripts in the new CHESS (Comprehensive Human Expressed SequenceS) database built from 9795 RNA-seq experiments, with comparisons to the RefSeq database. *ncRNA* noncoding RNA, *lncRNA* long noncoding RNA gene, *miscRNA* miscellaneous RNA

Type of gene	Number in RefSeq	Number in CHESS
Protein-coding genes	20,054	20,352
ncRNA genes		
- lncRNA	14,788	18,887
- Antisense	23	2144
- miscRNA	1217	1228
Total gene counts	36,082	42,611
Transcripts in protein-coding genes	127,718	266,331
Transcripts in ncRNA genes		
- lncRNA	28,015	49,892
- Antisense	28	2688
- miscRNA	2005	4347
Total transcripts	157,766	323,258

### Previously annotated proteins not observed in assembled GTEx transcripts

We analyzed the entire set of protein-coding genes in RefSeq to determine how many of them lacked support from any of the 9795 GTEx samples. We considered a gene to be supported if any GTEx transcript matched any of the gene’s exons; we did not require support for the precise exon-intron structure. Out of all 20,054 RefSeq genes, just 10 were not expressed in any of our samples (Table 2). We examined each of these 10 genes further and determined that five of them are likely to be errors in RefSeq, as we explain below. We deleted these five genes and their (five) transcripts from the CHESS gene set.

**Table 2:** Protein-coding genes from RefSeq that were not expressed in any of the 9795 RNA-seq samples from GTeX

NCBI gene ID	Gene name	Location	Product
101927562	LOC101927562	chr11 1554607–1556457	Uncharacterized^a^
101929097	LOC101929097	chr19 2511219–2513571	Uncharacterized^a^
107987231	LOC107987231	Chr16 29973622–29974648	Uncharacterized^a^
101928589	LOC101928589	chrX 110175773–110177788	Uncharacterized
728072	CT47A5	chrX 120963026–120966446	Cancer/testis antigen family 47 member A5
728049	CT47A8	chrX 120948422–120951842	Cancer/testis antigen family 47 member A8
728042	CT47A9	chrX 120943561–120946981	Cancer/testis antigen family 47 member A9
245927	DEFB113	chr6 49968677–49969625	Defensin beta 113
51206	GP6	Chr19 55013705–55038264	Glycoprotein VI platelet
102723822	LOC102723822 (GTPBP4/NGB)	Unplaced KI270752.1 8198–27137	Nucleolar GTP-binding protein 1-like

^a^These genes were removed from RefSeq by NCBI after publication of a preliminary version of these findings

The first four genes in Table 2—101927562, 101929097, 107987231, and 101928589—were predicted by computational pipelines at least 10 years ago. All loci are entirely contained in the 5′ UTRs of other well-characterized protein-coding genes. GenBank records indicate that the original computational predictions were based on EST evidence and on the presence of open reading frames, but no other evidence supports them. Their position in UTR regions explains the transcript (EST) evidence, but there is no reason to believe these are distinct protein-coding genes, and we did not include them in CHESS. (Note that the first three have recently been deleted from RefSeq.)

The next three genes in Table 2, CT47A5, CT47A8, and CT47A9, are genes that are normally expressed in germ cells and reactivated and expressed in some tumors [31]. Thus, it was not surprising that these genes were not expressed in the GTEx samples, which did not include either of these tissue types. Genes DEFB113 and GP6 both appear to be genuine. Both have multiple hits to other proteins, have known functions, and have strong experimental evidence supporting them. It is not clear why they were not present in the GTEx experiments, but it is possible they have highly tissue-specific expression.

Gene 102723822, the final entry in Table 2, is by far the most intriguing of the missing RefSeq proteins. This is a 14-exon gene with a well-characterized product (protein accession XP_006725006), with numerous orthologous proteins in other species. The protein resides on an unplaced scaffold (KI270752) in the current human reference genome, GRCh38. What is surprising about this protein is that its best alignments are to Chinese hamster (*Cricetulus griseus*) and other rodents. It is 98% identical to the hamster protein, but only 95% identical to the most similar human protein. It would be extraordinary for a human protein to have multiple hits to rodents that are all closer than any match to primates.

The KI270752 scaffold is 27,745 bp long, and upon investigation, we discovered that this scaffold is derived from a cosmid (accession AF065393) deposited in GenBank in 1998. The scaffold does not match any sequence on an alternate human assembly, CHM1_1.1 (GCA_000306695.2), which was built from whole-genome sequencing of a haploid cell line derived from a human hydatidiform mole. Given this evidence, we concluded that this unplaced scaffold represents contamination in the current human assembly. (Note: GenBank deleted this scaffold after learning of our findings.)

We also looked at protein-coding genes that were present in GENCODE but not RefSeq. In GENCODE release 25, we found 76 genes that were not in RefSeq of which 34 were not expressed in the GTEx experiments (Additional file 7). However, in GENCODE release 27, all but two of these 34 protein-coding genes were either deleted (27) or changed to noncoding (5), leaving just two genes (AP000351.1 and USP17L23) that were unique to GENCODE but not expressed in the GTEx data. Both of these genes are included in the CHESS catalog.

### Non-coding genes

From the complete GTEx data set, StringTie assembled a total of 30,467,424 transcripts, of which a majority (19,014,285; 62%) had only a single exon (Additional file 1: Table S1). 1,563,544 transcripts matched RefSeq or GENCODE entries, including 209,261 perfect matches and 1,354,283 partial matches. We retained all RefSeq and GENCODE transcripts as well as other transcripts for which we found protein-coding evidence, as described above. We then applied a series of filters to remove “noisy” transcripts from the remaining ones, as follows:

1. We required each transcript to be assembled in at least 10 samples, with an average TPM ≥ 1, or alternatively to have expression level as high as the outliers for known transcripts, defined as TPM > 13.87 (see Additional file 1).
2. We filtered out all single-exon noncoding transcripts.
3. We removed all transcripts that overlapped ribosomal RNA genes.
4. To avoid including pre-mRNA transcripts, we removed all transcripts that had retained introns, based on RefSeq and GENCODE intron annotations.
5. To eliminate pseudogenes, we filtered out any novel transcript that had at least 98% identity to a known transcript over 90% of its length. These transcripts might have been assembled from reads that originated from a real gene but that were aligned to a near-identical pseudogene copy of that gene.
6. We removed all transcripts that overlapped exons of annotated transcripts on the opposite strand, as well as transcripts that overlapped multiple known genes.
7. To reduce transcript assembly artifacts, we retained only the 10 most abundant novel transcripts at any given locus.
8. We discarded transcripts in loci corresponding to known processed pseudogenes or that overlapped immunoglobulin or T cell receptor segments.

After applying all the filters above and including the novel protein-coding transcripts described above, we were left with 116,186 transcripts that did not match any RefSeq or GENCODE transcripts. Of these, 96,382 represent isoforms (splice variants) of protein-coding genes, increasing the total number of protein-coding transcripts from 127,718 (in RefSeq) to 266,347 or 13.1 isoforms per protein-coding gene (Tables 1 and 3). 23,102 of the novel transcripts are also present in the FANTOM database, which used Cap Analysis of Gene Expression (CAGE), to create a large atlas of human genes with high-confidence 5′ ends [32]. Note that not all isoforms in protein-coding genes have an annotated ORF.

**Table 3:** Genes and transcripts in CHESS (v2.1) that are also found in either RefSeq (rel 108) or GENCODE (v27) (columns 2 and 5) and that are unique to CHESS (columns 3 and 6)

Gene biotype	Genes			Transcripts		
	Shared by RefSeq or GENCODE	Novel in CHESS	Novel + FANTOM	Shared by RefSeq or GENCODE	Novel in CHESS	Novel + FANTOM
Protein coding	20,128	224	26	169,959	96,372	23,102
LncRNA	16,216	2671	1407	34,222	15,670	5840
Antisense	598	1546	494	637	2051	606
MiscRNA	1227	1	1	2284	2063	476

The columns labeled “Novel + FANTOM” show the subset of CHESS genes and transcripts that are not found in RefSeq or GENCODE but that are present in the FANTOM gene catalog

The number of novel lncRNA gene loci remaining after these filtering steps was 4222, of which 1546 were antisense transcripts [33], which are contained within introns of other genes. Nearly half of the novel non-coding RNA genes (1902) were previously also found by the FANTOM consortium [32]. LncRNA genes have an average of ~ 2.6 isoforms in our catalog, although this number could increase if additional evidence emerges in the future.

Table 1 shows the number of the genes and transcripts, respectively, annotated as protein-coding and lncRNAs in CHESS and RefSeq. Additional file 1: Table S3 shows many of the other types of non-coding genes, in addition to lncRNAs, that are annotated in RefSeq. We should emphasize that the primary evidence of function for all transcripts unique to CHESS is their presence in the GTEx experimental data and that further evidence may be required to confirm their status as functional. Table 3 shows the number of genes and transcripts novel to CHESS, i.e., missing in both RefSeq and GENCODE. Note that RefSeq and GENCODE assign different biotypes to some transcripts that are present in both databases. For these transcripts, we used the RefSeq type for the CHESS entry.

### Intron comparisons

Related to the question of novel genes is the question of how many exons and introns are shared among CHESS, RefSeq, and GENCODE. Novel transcripts may in some cases represent novel combinations of exons—e.g., exon-skipping events—but in many cases, they include novel splice sites that create new exons and introns. To answer this question, we compared all of the protein coding and lncRNA transcripts in CHESS (version 2.1), RefSeq (release 108), and GENCODE (v28) to determine the number of (a) introns and (b) transcripts that were shared among all combinations of the three databases. For the CHESS introns and transcripts, we only considered those that were actually assembled by our pipeline for the purposes of this comparison, i.e., we did not include genes that were added to CHESS only because they were contained in one of the other databases.

**Fig. 2:**
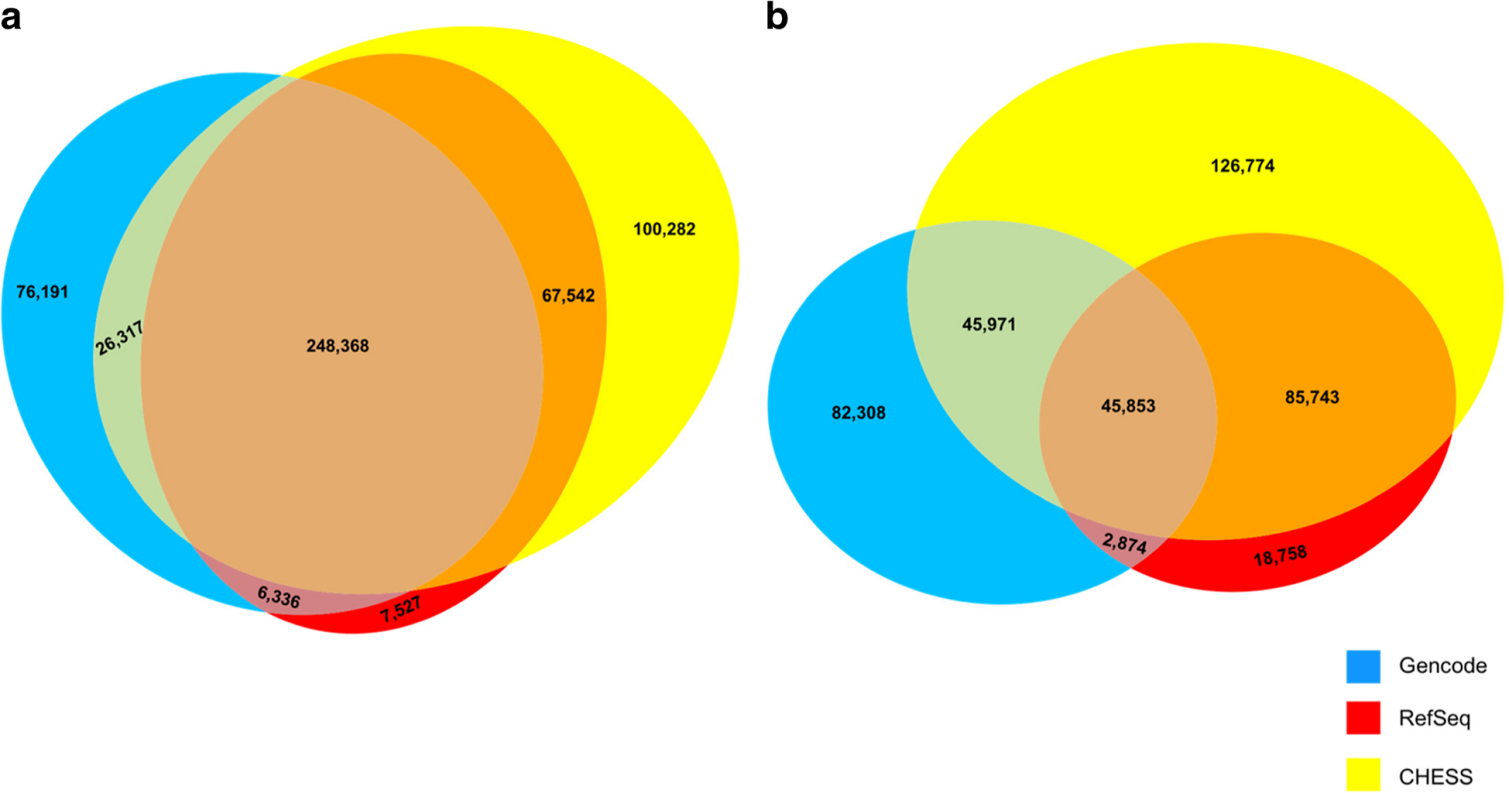
The number of **a** introns and **b** transcripts shared by and unique to all combinations of the CHESS (v2.1), RefSeq (rel 108), and GENCODE databases (v28). For this comparison, only transcripts and introns assembled directly by the CHESS pipeline were included. The CHESS database also includes additional transcripts that were added directly from RefSeq and GENCODE (see main text)

The results of these comparisons are shown in Fig. 2. Among the 533,563 introns contained in the union of the databases, 248,368 (47%) are shared among all three. 67,542 introns are shared by CHESS and RefSeq but missing from GENCODE, 26,317 are shared by CHESS and GENCODE but missing from RefSeq, and only 6336 are shared by RefSeq and GENCODE but missing from CHESS. As the figure shows, CHESS is in much closer agreement with GENCODE and Refseq than the two databases are with one another.

At the transcript level, the databases have much less overall agreement: out of 408,281 transcripts across all three databases, only 45,853 (11%) are shared (Fig. 2b). As with introns, CHESS agrees with the other two databases much more than they agree with each other: CHESS and RefSeq agree on 85,743 transcripts that are missing from GENCODE, while CHESS and GENCODE share 45,971 transcripts that are missing from RefSeq. In contrast, RefSeq and GENCODE share only 2874 transcripts that are missing from CHESS. Note here that transcripts were considered to be the same only if all introns matched exactly (see the “Methods” section). We conducted a similar comparison among the exons in all three databases (Additional file 1: Figure S12).

### Validation using differential expression

As an additional line of evidence that the novel genes in CHESS are functional, we analyzed the 9795 GTEx experiments to test whether any of the novel genes, both potentially protein coding and lncRNAs, were differentially expressed (DE). If a gene was expressed at significantly different levels—i.e., the transcription level of the gene differed between two conditions—then this finding would support (although not prove) the hypothesis that the gene is genuine.

**Fig. 3:**
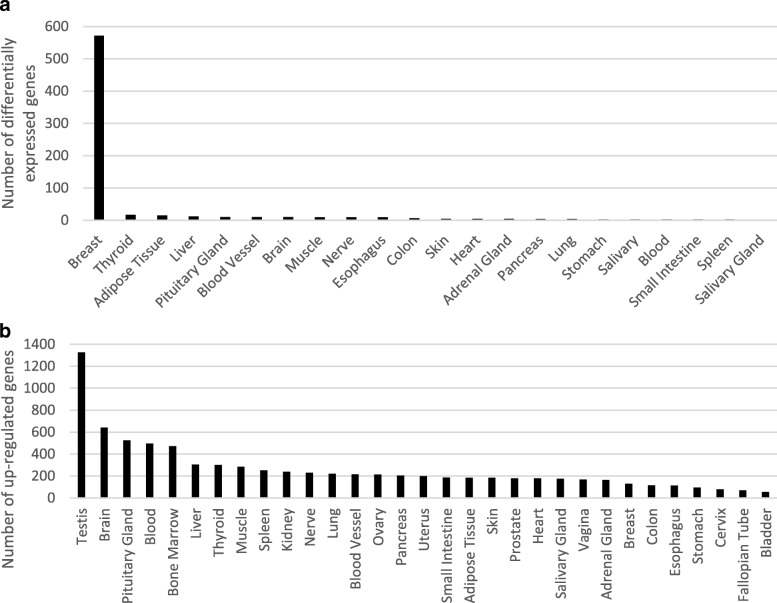
**a** The number of novel protein-coding and lncRNA genes that were differentially expressed between males and females, for each of the GTEx tissues that had both male and female samples. All tissues except kidney had at least 10 samples for each sex; kidney had 9 female and 29 male. **b** The number of novel protein-coding and lncRNA genes in CHESS that were upregulated in each of the 31 GTEx tissues as compared to the remaining tissues

We conducted two types of tests. First, we selected all tissues for which the GTEx data include both male and female samples (21 tissues) and computed which genes were differentially expressed between males and females (see the “Methods” section). A total of 608 novel CHESS genes, including 54 potentially protein-coding genes, were differentially expressed between the sexes (Fig. 3 and Additional file 8). Consistent with previously reported results [34], breast tissue showed far more DE genes than any other tissue.

Second, we evaluated all genes to determine how many were upregulated in at least one tissue (see the “Methods” section) and found that 2508 (86.6%) of the novel genes were upregulated (Fig. 3 and Additional file 9). By comparison, 89% of the RefSeq proteins and 87% of the RefSeq lncRNAs were upregulated in at least one tissue. Testis contained the largest number (1328) of novel upregulated genes (shown in Additional file 1: Table S5).

### Validation using mass spectrometry

One further possible line of evidence that a locus encodes a protein is direct evidence that the sequence is translated, which can be obtained from mass spectrometry experiments. Publications of two draft human proteomes have recently provided protein evidence for the majority of previously annotated protein-coding genes, in addition to some previously unknown proteins [35, 36]. These studies and others [37, 38] suggest that current reference annotation has not yet fully captured the protein-coding potential of the genome. To validate the coding potential of novel loci identified in this study, we searched the unmatched spectra from 30 human tissue/cell types (see the “Methods” section) against the novel predicted ORFs described in this study. Peptides identified in this search that were either identical to annotated proteins or mapped with a single mismatch were discarded. We manually examined the MS/MS spectra and discarded those with poor quality. We then created synthetic peptides corresponding to those that supported novel ORFs and compared the MS/MS spectra from synthetic peptides to experimental spectra.

**Fig. 4:**
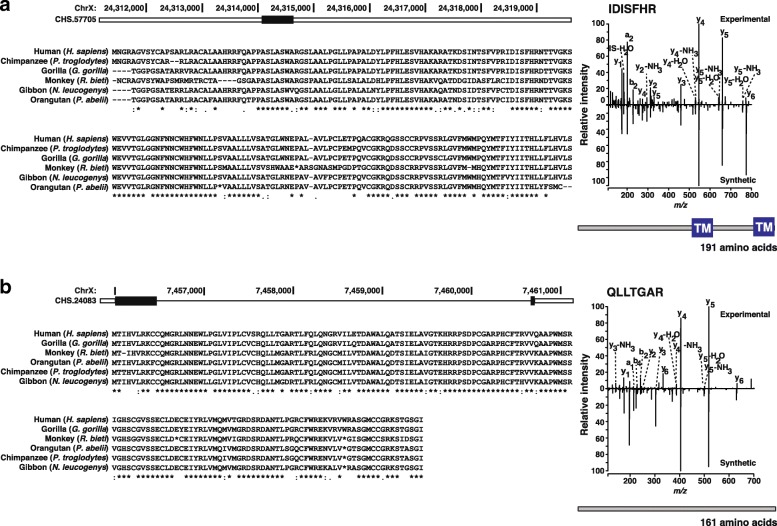
Multiple sequence alignments of novel CHESS protein-coding genes CHS.57705 (**a**) and CHS.24083 (**b**), each compared to five other primates, with annotated MS/MS spectra validating the identified peptides IDISFHR (**a**) and QLLTGAR (**b**) as shown on the right

Based on this analysis pipeline, we identified peptides that confirmed four of the novel protein-coding genes in the CHESS set. One example is CHS.57705, a transcript that encodes a 191 amino acid protein that has no similarity to known proteins but is conserved in other primates (Fig. 4a). This protein contains two transmembrane domains as predicted by SMART [39]. Another transcript, CHS.24083, encodes a protein of 161 amino acids without any predicted domains or similarity to known proteins (Fig. 4b), although it too is conserved in primates. Additional file 1: Table S6 shows all four novel ORFs identified with peptide evidence from proteomics data analysis. Additional file 1: Figure S9 shows the two additional cases where the mass spectra from synthetic peptides validated the experimental spectra as well as two cases (neither of which passed all the filters required to be a CHESS gene) that were not validated. We note that the abundance of these novel transcripts was very low and the ORFs are relatively short, both of which may explain the small number of identified peptides.

### Methods

The initial GTEx data release contained 1641 RNA-seq samples [20, 40], and a subsequent publication described a much larger set of 8555 samples collected from 40 body sites [41]. Our data represents a later GTEx data release with 9795 samples across 31 tissue types and 54 body sites, summarized in Additional file 1: Table S2.

#### Alignment and assembly

**Fig. 5:**
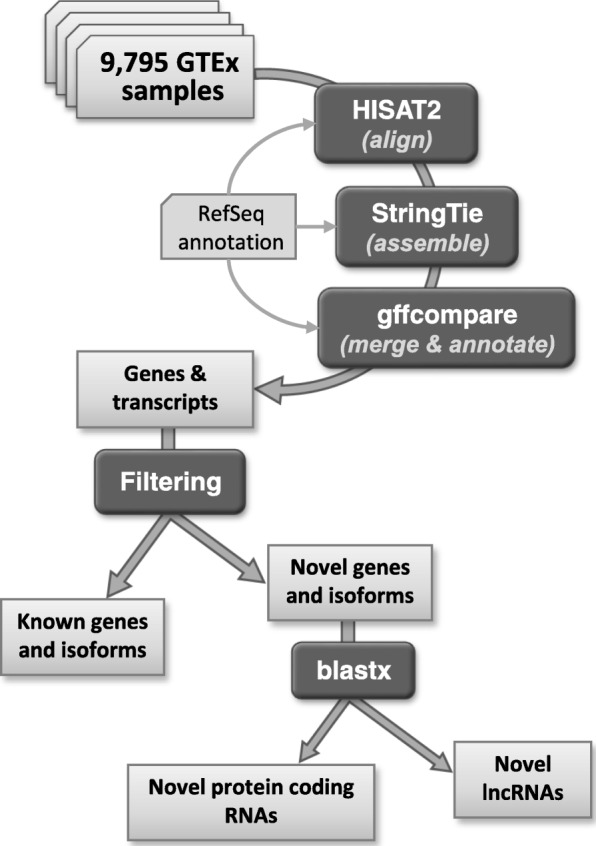
Summary of the computational pipeline used to align and assemble all 9795 RNA-seq samples

In total, the 9795 RNA-seq samples contain 899,960,113,026 reads (449,980,056,513 pairs), an average of 91.9 million reads (46 M pairs) per sample. The RNA-seq assembly process, illustrated in Fig. 5, required multiple steps of alignment, assembly, and quantification [42] for each of the samples. We aligned each sample to release GRCh38.p8 of the human genome using HISAT2 [43] (http://ccb.jhu.edu/software/hisat2) with default parameters, providing it with the RefSeq annotation. We then assembled the alignments using StringTie [44] (https://github.com/gpertea/stringtie) again providing the RefSeq annotation. Both HISAT2 and StringTie use annotation as a guide when provided, but both programs find novel splice sites (HISAT) and novel transcripts (StringTie) whenever necessitated by the data. The RefSeq annotation provided here contained 20,054 protein-coding genes, 15,779 long noncoding RNA (lncRNA) genes, 16,131 pseudogenes, and 629 tRNA genes, as well as a few other specialized categories of annotation (Additional file 1: Table S3).

#### Timings

Alignment of reads with HISAT2 was the most computationally intensive step in the process. On average, 88.3% of reads aligned successfully across all 9795 samples. The alignment steps included uncompressing the original SRA files, aligning them to the genome with HISAT2 (using eight CPUs in parallel), and compressing the output to produce BAM files. These steps took an average of 43 min per file (sample), using a 32-core server with a 2.13 GHz Intel Xeon E7 for benchmarking. Following alignment, the aligned reads were sorted and converted to the compact CRAM format. This process took an average of 17 min per sample using eight threads. Assembly and quantification with StringTie took an average of 24 min per sample using four threads. Thus, the total average time to process one sample, with much of the time limited by I/O speed for the very large files involved, was 84 min. Processing all 9795 samples required about 13,700 h (571 days); by dividing the computation across many processors, this was reduced to about 30 days total elapsed time. Note that attempts to parallelize this process further would require distributing the files across many independent storage units; otherwise, contention for file access would make parallel processing ineffective.

After the initial assembly steps, many transcript assemblies were fragmented (i.e., not full-length) due to low coverage in particular samples. To correct this problem, we compared all transcripts and transcript fragments across all samples and merged any transcripts that were contained within or overlapped others. For this merging step, we first used the program gffcompare (ccb.jhu.edu/software/stringtie/gff.shtml) to merge all GTF (Gene Transfer Format) files from the original samples on a tissue-by-tissue basis. Following this step, which produced a single GTF file for each of the 31 tissues, we merged the 31 files together to produce a single, consistent set of transcripts that accounted for all samples.

We computed expression levels using both TPM (transcripts per million reads) and FPKM (fragments per kilobase of exon per million reads). Additional file 1 shows the distributions of expression levels for all genes in RefSeq, including distributions used to calculate outliers for protein coding and lncRNA genes (Additional file 1: Figures S1–S5). For consistency, we used TPM values as thresholds for all filtering steps.

We identified the longest open reading frames (ORFs) in transcripts with gffread (github.com/gpertea/gffread). We ran BLAST searches of all ORFs against the Swiss-Prot section of UniProt (release 2017_09) and the *nr* database, a comprehensive, non-redundant protein database downloaded from NCBI in February 2017. The mammalian protein database used in some searches was a subset of nr. When considering lncRNA matches to proteins from this database, we considered a protein to have unknown function if its name included any of the following keywords: hypothetical, unnamed protein product, uncharacterized protein, unknown, pseudogene, LOC, PRO, orf, or open reading frame. We also excluded proteins whose only annotation was a name with the prefix hCG, which are computational predictions based only on de novo gene finding programs and/or EST evidence reported in one of the original human genome papers [7]. We identified known protein domains in the ORFs by searching the translated sequences against the Pfam protein family database release 31.0 [27] using HMMER’s hmmscan v3.2.1 [45]. The multiple sequence alignments shown in Fig. 1 and Additional file 1: Figures S6–S8 were produced with SeaView v4.6.2 [46].

#### Comparisons to known annotation databases

We used gffcompare to compare assembled transcripts to the RefSeq and GENCODE databases. We downloaded the FANTOM transcripts defined as “robust” from fantom.gsc.riken.jp/5/suppl/Hon_et_al_2016/data/assembly/lv3_robust/ and used the UCSC’s liftOver program with the default parameters to remap them from GRCh37 to GRCh38. We checked all ~ 30 million StringTie-assembled transcripts against these remapped FANTOM transcripts using the trmap program (github.com/gpertea/trmap), a specialized version of gffcompare, optimized for streaming a large set of transcripts against another set, for the purpose of reporting and classifying overlaps between them. Because the FANTOM transcripts had experimental data to support their 5′ ends, we adjusted the ends of the CHESS transcripts when they otherwise matched the full length of a FANTOM transcript.

#### Intron and transcript comparisons

We used a custom program to generate and compare lists of introns in RefSeq (release 108), GENCODE (v28), and CHESS (v2.1). After excluding introns in CHESS that we did not assemble through our pipeline, we counted the number of introns shared in all combinations of the databases. Introns were considered the same only if their start and end coordinates matched exactly. To compare transcripts in all three databases, we modified our program to generate a list of intron chains for each transcript. We considered a transcript to be the same only if the intron structure matched exactly, i.e., the transcripts contained exactly the same introns.

#### Differential expression between sexes

We used Salmon [47] to generate quantification estimates of the complete set of CHESS transcripts assembled from the GTEx data. The advantages to using Salmon over other transcript quantification programs include speed, compatibility with downstream analysis tools, and the ability to retain multi-mapped reads. Salmon relies on the use of an index built from transcript sequences to quasi-map RNA-seq reads in the quantification step. To obtain these transcript sequences, we used gffread (http://ccb.jhu.edu/software/stringtie/gff.shtml) to extract them from the CHESS GFF file. The index built from the resulting multi-fasta file and the raw sequencing reads were then used to generate CHESS transcript abundance estimates for each GTEx sample.

We used the tximport package [48] to import the Salmon output and generate separate gene-level count matrices for each tissue that contained both male and female samples. To account for the widely varying number of samples per tissue, we chose a random subset of samples from tissues with large numbers of samples.

We then used the resulting count matrices as input to DESeq2 [49] to conduct differential expression analysis within each of the 31 tissues independently, comparing male to female samples. We used the false discovery rate (FDR) computed from DESeq2’s implementation of the Benjamini-Hochberg adjustment. The set of differentially expressed genes with FDR < 0.05 was then filtered to extract those that were exclusive to CHESS.

From the results of the 31 DESeq2 experiments, we created a single list of genes differentially expressed in at least one tissue. For breast tissue, we counted all genes with an FDR < 0.05. For each additional tissue, we only included genes with an FDR < 0.002 to correct for multiple comparisons across tissues. An FDR threshold of 0.002 for each gene in each tissue corresponds to an FDR of ~ 0.05 for each gene across all tissues. The major differences between male and female breast tissue, as found in multiple previous studies, led us to expect a large number of differentially expressed genes for that tissue type.

#### Tissue-specific differential expression

We started by randomly selecting 20 samples from each of the 31 tissues. In cases where the given tissue had fewer than 20 samples, we selected all samples. Using tximport, we then created one gene-level count matrix for these 591 samples. With this count matrix, we ran DESeq2 to test for differential expression between tissues while controlling for the effect of sex. Using the “contrast” argument of the results function in DESeq2, we made 31 different comparisons to find genes upregulated in each tissue. Each comparison contrasted the gene expression in the tissue of interest to the average expression across all other tissues. For each tissue, we considered upregulated genes with an FDR < 0.05 significant and then filtered this list to report only novel, protein-coding genes. To create a list of all novel genes upregulated in at least one tissue, we reduced the FDR threshold to 0.0015 to correct for the 31 comparisons. An FDR threshold of 0.0015 for each gene in each tissue results in a (conservative) FDR of ~ 0.05 for each gene across all tissues.

#### Mass spectrometry

Unmatched MS/MS spectra from a previous study [35] were searched against translated products of predicted CHESS ORFs using the SEQUEST search engine on Proteome Discoverer 2.1 software platform (Thermo Fisher Scientific). Carbamidomethylation of cysteine and oxidation of methionine were specified as fixed and variable modifications. Mass tolerance limits were set to 10 ppm and 0.02 Da for precursor and fragment ions, respectively. A target-decoy database approach was employed to filter the identified peptides at a 1% false discovery rate. Peptide sequences that corresponded to novel genes were synthesized (JPT Peptide Technologies, Berlin, Germany), analyzed on an Orbitrap Fusion Lumos Tribrid mass spectrometer (Thermo Fisher), and compared against the experimental spectra. Putative translational products of novel ORFs were aligned using BLAST against the NCBI nr protein database, and domain prediction was carried out using SMART [39]. Multiple sequence alignment of protein sequences was performed using Clustal Omega [50].

## Discussion

The new human gene catalog described here, CHESS, contains an inclusive set of genes based on nearly 10,000 RNA sequencing experiments. As such, it provides a reference with substantially greater experimental support than previous human gene catalogs. Although it represents only a modest increase in the number of potential protein-coding genes (224, or 1.1% of the 20,352 total), it more than doubles the number of splice variants and other isoforms of these genes, to 266,331 (Table 1). This more comprehensive catalog of genes and splice variants should provide a better foundation for RNA-seq experiments, exome sequencing experiments, genome-wide association studies, and many other studies that rely on human gene annotation as the basis for their analysis.

We produced the novel genes and transcripts in CHESS using a genome-guided assembly pipeline including HISAT2 and StringTie. Although this pipeline is among the most accurate approaches for novel transcriptome discovery [44, 51], transcript mis-assemblies may still be present in our catalog due to several factors that negatively affect the reconstruction process, including sequencing errors, incomplete coverage of transcripts, and algorithmic assumptions. Assembly errors are most likely to occur for transcripts that have low coverage, hence our requirement that all novel transcripts be assembled in at least 10 samples with an average TPM > 1.

Given the history of changes in our knowledge of human genes and transcripts, it seems highly likely that this new database will change further in the future. In particular, many of the more than 18,000 noncoding RNA genes have less evidential support than the protein-coding component of the genome, and this number may decline over time just as the human gene count declined from 2001 to the present. The CHESS database of genes and transcripts, which is freely available at *http://ccb.jhu.edu/chess*, will be updated over time as new evidence emerges.

### Transcriptional noise

Perhaps the most striking result of this study is the vast number of transcripts that appear to have no function at all. Across all data sets and all tissue types, we observed over 30 million distinct transcripts in approximately 700,000 distinct genomic locations, of which only about 42,000 (6%) appear to represent functional gene loci. As others have argued [22], the mere fact that a sequence is transcribed is insufficient evidence to conclude that it is a gene, despite the fact that early genomics studies made precisely that assumption. It appears instead that 95% of the transcribed locations in the human genome are merely transcriptional noise, explained by the nonspecific binding of RNA polymerase to random or very weak binding sites in the genome. This observation is consistent with efforts to identify sequence motifs that signal the initiation of transcription, which have largely failed because no highly conserved sequences exist.

Similarly, the vast majority of the transcript variants themselves also appear nonfunctional. Although this study greatly increases the number of isoforms of known genes, the 323,258 transcripts reported here represent just 1.1% of the 30,467,424 distinct transcripts observed across all 9795 data sets. This suggests that the splicing machinery too, like RNA polymerase, is highly nonspecific in its actions, in agreement with previous studies that found that the vast majority of observed splice variants correspond to errors [52]. The splice sites themselves are much better conserved than any transcription initiation site, but the cellular machinery for cutting and pasting the exons together appears to be inefficient, producing many variations that are simply non-functional, with low-abundance isoforms being especially likely to be the result of errors [53]. It is possible that our criteria for excluding a transcript were too strict, but even so, it seems unlikely that a large proportion of the transcripts we rejected are essential for the cell.

Note that functional transcripts occur at much higher abundances than non-functional ones, as shown in Additional file 1: Figures S2–S5. If we add up the expression levels of all the functional transcripts and compare that to the total expression of non-functional transcripts, we find that 68% of the transcriptional activity is devoted to producing functional transcripts, while 32% is apparently spent (and presumably wasted) on nonfunctional ones. Thus, although the sheer amount of variation is very large, about two thirds of the RNA molecules in the cell are functional.

The overall picture that emerges from this analysis is that the cell is a relatively inefficient machine, transcribing more DNA into RNA than it needs. Ever since the discovery of introns [54, 55], we have known that genomes contain large regions that appear to have no function. Based on the results described here, it appears that nearly 99% of the transcriptional variety produced in human cells has no apparent function, although most of these variants appear at such low levels that they cumulatively account for only 32% of transcriptional activity.

## Additional files

Additional file 1: Additional text, tables (**Tables S1**–**S6**), and figures (**Figures S1**–**S11**) supporting the main analyses. (DOCX 4942 kb)

Additional file 2: Novel CHESS protein-coding transcripts with Pfam domain hits. (XLSX 19 kb)

Additional file 3: Novel CHESS protein-coding transcripts with CDD domain hits. (XLSX 56 kb)

Additional file 4: List of all novel potential protein-coding genes in CHESS. (XLSX 63 kb)

Additional file 5: List of all potential protein-coding transcripts from the novel genes in CHESS. (XLSX 80 kb)

Additional file 6: List of protein-coding genes present in GENCODE release 25 that were either deleted or overlapped genes with the type “non-coding” in GENCODE release 27. (XLSX 12 kb)

Additional file 7: Protein coding genes in GENCODE release 25 that were not expressed in any of the GTEx experiments. (XLSX 10 kb)

Additional file 8: Novel protein-coding and lncRNA genes in CHESS that are differentially expressed between males and females. Comparisons were conducted in all tissues for which both male and female samples were available. (XLSX 33 kb)

Additional file 9: All novel protein-coding and lncRNA genes in CHESS that were upregulated in at least one tissue. Shown are the tissues in which upregulation was observed. (XLSX 89 kb)

## References

[1] Vogel F (1964). A preliminary estimate of the number of human genes. Nature.

[2] Schuler GD, Boguski MS, Stewart EA, Stein LD, Gyapay G, Rice K, White RE, Rodriguez-Tome P, Aggarwal A, Bajorek E (1996). A gene map of the human genome. Science.

[3] Antequera F, Bird A (1994). Predicting the total number of human genes. Nat Genet.

[4] Fields C, Adams MD, White O, Venter JC (1994). How many genes in the human genome?. Nat Genet.

[5] Liang F, Holt I, Pertea G, Karamycheva S, Salzberg SL, Quackenbush J (2000). Correction: gene index analysis of the human genome estimates approximately 120,000 genes. Nat Genet.

[6] The International Human Genome Sequencing Consortium (2001). Initial sequencing and analysis of the human genome. Nature.

[7] Venter JC, Adams MD, Myers EW, Li PW, Mural RJ, Sutton GG, Smith HO, Yandell M, Evans CA, Holt RA (2001). The sequence of the human genome. Science.

[8] International Human Genome Sequencing Consortium (2004). Finishing the euchromatic sequence of the human genome. Nature.

[9] Clamp M, Fry B, Kamal M, Xie X, Cuff J, Lin MF, Kellis M, Lindblad-Toh K, Lander ES (2007). Distinguishing protein-coding and noncoding genes in the human genome. Proc Natl Acad Sci U S A.

[10] Ezkurdia I, Juan D, Rodriguez JM, Frankish A, Diekhans M, Harrow J, Vazquez J, Valencia A, Tress ML (2014). Multiple evidence strands suggest that there may be as few as 19,000 human protein-coding genes. Hum Mol Genet.

[11] Pertea M, Salzberg SL (2010). Between a chicken and a grape: estimating the number of human genes. Genome Biol.

[12] O'Leary NA, Wright MW, Brister JR, Ciufo S, Haddad D, McVeigh R, Rajput B, Robbertse B, Smith-White B, Ako-Adjei D (2016). Reference sequence (RefSeq) database at NCBI: current status, taxonomic expansion, and functional annotation. Nucleic Acids Res.

[13] Harrow J, Frankish A, Gonzalez JM, Tapanari E, Diekhans M, Kokocinski F, Aken BL, Barrell D, Zadissa A, Searle S (2012). GENCODE: the reference human genome annotation for The ENCODE Project. Genome Res.

[14] Farrell CM, O'Leary NA, Harte RA, Loveland JE, Wilming LG, Wallin C, Diekhans M, Barrell D, Searle SM, Aken B (2014). Current status and new features of the Consensus Coding Sequence database. Nucleic Acids Res.

[15] Need AC, Shashi V, Hitomi Y, Schoch K, Shianna KV, McDonald MT, Meisler MH, Goldstein DB (2012). Clinical application of exome sequencing in undiagnosed genetic conditions. J Med Genet.

[16] Zhu X, Petrovski S, Xie P, Ruzzo EK, Lu YF, McSweeney KM, Ben-Zeev B, Nissenkorn A, Anikster Y, Oz-Levi D (2015). Whole-exome sequencing in undiagnosed genetic diseases: interpreting 119 trios. Genet Med.

[17] Guttman M, Amit I, Garber M, French C, Lin MF, Feldser D, Huarte M, Zuk O, Carey BW, Cassady JP (2009). Chromatin signature reveals over a thousand highly conserved large non-coding RNAs in mammals. Nature.

[18] Cabili MN, Trapnell C, Goff L, Koziol M, Tazon-Vega B, Regev A, Rinn JL (2011). Integrative annotation of human large intergenic noncoding RNAs reveals global properties and specific subclasses. Genes Dev.

[19] Kung JT, Colognori D, Lee JT (2013). Long noncoding RNAs: past, present, and future. Genetics.

[20] The GTEx Consortium (2015). Human genomics. The Genotype-Tissue Expression (GTEx) pilot analysis: multitissue gene regulation in humans. Science.

[21] Adams MD, Kerlavage AR, Fields C, Venter JC (1993). 3,400 new expressed sequence tags identify diversity of transcripts in human brain. Nat Genet.

[22] Palazzo AF, Lee ES (2015). Non-coding RNA: what is functional and what is junk?. Front Genet.

[23] Raj A, Peskin CS, Tranchina D, Vargas DY, Tyagi S (2006). Stochastic mRNA synthesis in mammalian cells. PLoS Biol.

[24] Trapnell C, Williams BA, Pertea G, Mortazavi A, Kwan G, van Baren MJ, Salzberg SL, Wold BJ, Pachter L (2010). Transcript assembly and quantification by RNA-Seq reveals unannotated transcripts and isoform switching during cell differentiation. Nat Biotechnol.

[25] Trapnell C, Roberts A, Goff L, Pertea G, Kim D, Kelley DR, Pimentel H, Salzberg SL, Rinn JL, Pachter L (2012). Differential gene and transcript expression analysis of RNA-seq experiments with TopHat and Cufflinks. Nat Protoc.

[26] Altschul SF, Madden TL, Schaffer AA, Zhang J, Zhang Z, Miller W, Lipman DJ (1997). Gapped BLAST and PSI-BLAST: a new generation of protein database search programs. Nucleic Acids Res.

[27] Finn RD, Coggill P, Eberhardt RY, Eddy SR, Mistry J, Mitchell AL, Potter SC, Punta M, Qureshi M, Sangrador-Vegas A (2016). The Pfam protein families database: towards a more sustainable future. Nucleic Acids Res.

[28] Marchler-Bauer A, Bo Y, Han L, He J, Lanczycki CJ, Lu S, Chitsaz F, Derbyshire MK, Geer RC, Gonzales NR (2017). CDD/SPARCLE: functional classification of proteins via subfamily domain architectures. Nucleic Acids Res.

[29] Szmulewicz MN, Novick GE, Herrera RJ (1998). Effects of Alu insertions on gene function. Electrophoresis.

[30] Hancks DC, Kazazian HH (2010). SVA retrotransposons: evolution and genetic instability. Semin Cancer Biol.

[31] Chen YT, Iseli C, Venditti CA, Old LJ, Simpson AJ, Jongeneel CV (2006). Identification of a new cancer/testis gene family, CT47, among expressed multicopy genes on the human X chromosome. Genes Chromosomes Cancer.

[32] Hon CC, Ramilowski JA, Harshbarger J, Bertin N, Rackham OJ, Gough J, Denisenko E, Schmeier S, Poulsen TM, Severin J (2017). An atlas of human long non-coding RNAs with accurate 5′ ends. Nature.

[33] Mercer TR, Dinger ME, Sunkin SM, Mehler MF, Mattick JS (2008). Specific expression of long noncoding RNAs in the mouse brain. Proc Natl Acad Sci U S A.

[34] Mele M, Ferreira PG, Reverter F, DeLuca DS, Monlong J, Sammeth M, Young TR, Goldmann JM, Pervouchine DD, Sullivan TJ (2015). Human genomics. The human transcriptome across tissues and individuals. Science.

[35] Kim MS, Pinto SM, Getnet D, Nirujogi RS, Manda SS, Chaerkady R, Madugundu AK, Kelkar DS, Isserlin R, Jain S (2014). A draft map of the human proteome. Nature.

[36] Wilhelm M, Schlegl J, Hahne H, Gholami AM, Lieberenz M, Savitski MM, Ziegler E, Butzmann L, Gessulat S, Marx H (2014). Mass-spectrometry-based draft of the human proteome. Nature.

[37] Na CH, Barbhuiya MA, Kim MS, Verbruggen S, Eacker SM, Pletnikova O, Troncoso JC, Halushka MK, Menschaert G, Overall CM, Pandey A (2018). Discovery of noncanonical translation initiation sites through mass spectrometric analysis of protein N termini. Genome Res.

[38] Samandi S, Roy AV, Delcourt V, Lucier JF, Gagnon J, Beaudoin MC, Vanderperre B, Breton MA, Motard J, Jacques JF, et al. Deep transcriptome annotation enables the discovery and functional characterization of cryptic small proteins. eLife. 2017;6:e27860. 10.7554/eLife.27860. 10.7554/eLife.27860 PMC5703645 29083303

[39] Letunic I, Bork P (2018). 20 years of the SMART protein domain annotation resource. Nucleic Acids Res.

[40] Carithers LJ, Ardlie K, Barcus M, Branton PA, Britton A, Buia SA, Compton CC, DeLuca DS, Peter-Demchok J, Gelfand ET (2015). A novel approach to high-quality postmortem tissue procurement: the GTEx project. Biopreserv Biobank.

[41] Wheeler HE, Shah KP, Brenner J, Garcia T, Aquino-Michaels K, Consortium GT, Cox NJ, Nicolae DL, Im HK (2016). Survey of the heritability and sparse architecture of gene expression traits across human tissues. PLoS Genet.

[42] Pertea M, Kim D, Pertea GM, Leek JT, Salzberg SL (2016). Transcript-level expression analysis of RNA-seq experiments with HISAT, StringTie and Ballgown. Nat Protoc.

[43] Kim D, Langmead B, Salzberg SL (2015). HISAT: a fast spliced aligner with low memory requirements. Nat Methods.

[44] Pertea M, Pertea GM, Antonescu CM, Chang TC, Mendell JT, Salzberg SL (2015). StringTie enables improved reconstruction of a transcriptome from RNA-seq reads. Nat Biotechnol.

[45] Eddy SR (2011). Accelerated profile HMM searches. PLoS Comput Biol.

[46] Gouy M, Guindon S, Gascuel O (2010). SeaView version 4: a multiplatform graphical user interface for sequence alignment and phylogenetic tree building. Mol Biol Evol.

[47] Patro R, Duggal G, Love MI, Irizarry RA, Kingsford C (2017). Salmon provides fast and bias-aware quantification of transcript expression. Nat Methods.

[48] Soneson C, Love MI, Robinson MD (2015). Differential analyses for RNA-seq: transcript-level estimates improve gene-level inferences. F1000Res.

[49] Love MI, Huber W, Anders S (2014). Moderated estimation of fold change and dispersion for RNA-seq data with DESeq2. Genome Biol.

[50] Sievers F, Wilm A, Dineen D, Gibson TJ, Karplus K, Li W, Lopez R, McWilliam H, Remmert M, Soding J (2011). Fast, scalable generation of high-quality protein multiple sequence alignments using Clustal Omega. Mol Syst Biol.

[51] Voshall Adam, Moriyama Etsuko N. (2018). Next-Generation Transcriptome Assembly: Strategies and Performance Analaysis. Bioinformatics in the Era of Post Genomics and Big Data.

[52] Saudemont B, Popa A, Parmley JL, Rocher V, Blugeon C, Necsulea A, Meyer E, Duret L (2017). The fitness cost of mis-splicing is the main determinant of alternative splicing patterns. Genome Biol.

[53] Pickrell JK, Pai AA, Gilad Y, Pritchard JK (2010). Noisy splicing drives mRNA isoform diversity in human cells. PLoS Genet.

[54] Chow LT, Gelinas RE, Broker TR, Roberts RJ (1977). An amazing sequence arrangement at the 5′ ends of adenovirus 2 messenger RNA. Cell.

[55] Berget SM, Moore C, Sharp PA (1977). Spliced segments at the 5′ terminus of adenovirus 2 late mRNA. Proc Natl Acad Sci U S A.

[56] Pertea M, Shumate A, Pertea G, Varabyou A, Breitwieser FP, Salzberg SL (2018). CHESS: a new human gene catalog curated from thousands of large-scale RNA sequencing experiments reveals extensive transcriptional noise.

