# Supplementary material for: CHESS: a new human gene catalog curated from thousands of large-scale RNA sequencing experiments reveals extensive transcriptional noise
Source: Genome Biol. 2018 Nov 28;19:208. doi: 10.1186/s13059-018-1590-2 (PMC6260756; doi:10.1186/s13059-018-1590-2)
Supplement: Supplementary file 1 — Additional text, tables (Tables S1–S6), and figures (Figures S1–S11) supporting the main analyses. (DOCX 4942 kb) [file 13059_2018_1590_MOESM1_ESM.docx]

Supplementary materials for *“Thousands of large-scale RNA sequencing experiments yield a new human gene list and reveal extensive transcriptional noise”*

Mihaela Pertea, Alaina Shumate, Geo Pertea, Ales Varabyou, Florian P. Breitwieser, Yu-Chi Chang, Anil K. Madugundu, Akhilesh Pandey, and Steven L. Salzberg

**Supplementary Methods, Tables, and Figures**

**The CHESS database**

The CHESS database (http://ccb.jhu.edu/chess) contains all genes and transcripts in a set of tables in genome feature format (GFF), including: (1) a GFF file containing all the genes and transcripts described in this study, (2) a GFF file containing these genes plus the tRNA and rRNA genes from RefSeq, and (3) a GFF file containing all the CHESS genes plus all other feature types in RefSeq, shown in Table S3 below. Note that GRCh38 has hundreds of alternative scaffolds (labelled "alt") and patches, and many RefSeq genes occur on both the primary chromosomes and these alternative copies. The genes themselves are not truly duplicated, but rather appear on portions of the GRCh38 assembly that are represented in more than one version. As a result, some genes occur multiple times in the GFF files, each time with a distinct GFF ID, but with a single unique gene ID that can be found in the comments field. We counted each of these genes only once in our total gene counts. In order to provide a clean gene set, our catalog also provides a separate table with just one line for each gene in CHESS, listing the gene's location, identifiers, description (if available), and other information. Genes with multiple identifiers due to their presence on alternative assemblies occur just once in this table.

**Features of intergenic transcripts**

From the entire set of assembled transcripts across all GTEx samples, there are 5,081,171 intergenic transcripts in 668,018 loci that do not overlap any annotated genes in RefSeq, Gencode, or Ensembl. The vast majority of these are single-exon genes: only 139,289 transcripts in 41,979 loci have more than one exon. Out of these multi-exon transcripts, 43,512 in 16,556 loci appear in 2 or more samples.

Following is a summary of how many transcripts satisfied the series of filtering criteria used to identify potential protein-coding genes from among these previously unidentified loci.

1. The transcript was either a multi-exon transcript with an expression value of at least 1 TPM, or a single exon transcript with a high expression value, defined as an outlier (see Figure S5) with a TPM>13.87. These criteria were satisfied by 85,115 transcripts in 25,682 loci.
2. The length of the open reading frame (ORF) must be at least 60 amino acids. 41,602 transcripts remained after this step.
3. The ORF could not overlap known LINE or LTR repeat elements, or overlap ribosomal RNA genes. 22,544 transcripts remained after this step.
4. The BLAST e-value of the best mammalian or SwissProt protein alignment must be 10^-15^ or smaller. This step left 5,014 transcripts.
5. When the protein matched another human protein, the length of the ORF should be at least 75% of the other protein's length (in order to eliminate pseudogenes). This step left 2,841 transcripts.
6. If a transcript was contained in another transcript, it had to have a higher TPM than the containing transcript. This step left 1334 transcripts (in 1,177 genes). Of these, 654 transcripts had their best match to a non-human protein in either **nr** or Swiss-Prot.

Supplementary **Table S1** displays the number of transcripts that had 1, 2, 3, etc. exons across all samples. **Table S2** shows the 54 body sites represented in the GTEx data and the number of RNA-seq samples from each body site.

**ORF comparisons.** To examine the coding potential of novel isoforms that were added to known protein-coding genes, we verified the compatibility of novel exon chains with the previously annotated open reading frames (ORFs). From 16,496 multi-exon loci found in either RefSeq or Gencode (or both), we identified 150,111 isoforms previously annotated in either RefSeq or Gencode, and 95,974 additional isoforms that are unique to CHESS. Of the 150,111 "known" isoforms, 113,402 contained an annotation of a protein coding sequence (CDS).

From the 95,974 novel isoforms, we found that 51,831 (54%) are compatible with at least one CDS from the previously annotated genes; i.e., the new isoform maintains the coding sequence, possibly with an insertion or deletion that maintains the reading frame. 39,242 (41%) of the novel transcripts preserved the peptide chain completely.

For comparison, we applied the same method to the 150,111 known RefSeq and Gencode transcripts. Of these, 110,032 (73%) isoforms are compatible with at least one other coding-sequence from the same locus without a frameshift, and 85,711 (57%) isoforms preserved the full protein sequence without modifications.

*Methods for ORF comparisons*. We developed a custom Python program to evaluate the differences between amino acid sequences encoded by protein-coding isoforms of the same gene. For each previously annotated gene with at least one CDS and at least one novel transcript, we first computed the Cartesian product between the set of all novel transcripts and the set of all CDS annotations. We then proceeded to compute a set of all indel alterations required to accommodate an annotated CDS into a suggested exon chain by finding all intervals that were spanned by either member of the pair but not both. For each evaluated exon chain, only the best matching CDS was reported along with any alterations observed for that match. We then used the length of individual intervals to identify both frameshifting and non-frameshifting mutations. We used the position of the intervals with respect to the known CDS to identify cases where the protein sequence would be truncated on either end. We applied the same method to identify differences in protein sequences among previously annotated isoforms of protein-coding genes.

**Distributions of expression levels for RefSeq genes.** We computed TPM (transcripts per million) values for all transcripts that corresponded to RefSeq genes in the categories of protein coding genes, lncRNA genes, and pseudogenes. **Table S3** summarizes how many of these genes and other features are in the RefSeq annotation. Because the current human reference genome has hundreds of alternative scaffolds, the number of genes in Table S3 does not correspond precisely to the number of loci where a particular gene is present. For example, the protein-coding gene KIR3DL3 is present in 47 loci: one locus on the Primary Assembly, 14 on patches, and 32 on alternative scaffolds. In total, the 20,054 RefSeq protein-coding genes occur in 22,554 loci in the GRCh38.p8 assembly, as shown in **Table S4**.

In order to compute accurate statistics on average expression levels for all genes in this study, we identified a subset of genes that occur in just a single locus (i.e., single-copy genes). For each protein coding gene and lncRNA we extracted all transcripts from RefSeq (GRCh38.p8) that satisfied the following conditions:

1. *protein-coding* *transcripts* had to be labeled as mRNA;
2. *lncRNA transcripts* had to be labeled as lncRNA in the annotation and belong to a gene annotated with the ncRNA biotype;
3. transcripts were not annotated as partial;
4. transcripts did not have the attribute ‘pseudo=true’; and
5. transcripts did not come from genes with multiple loci in the genome (such as genes present on both the main chromosomes and on the alternate scaffolds).

The total number of RefSeq transcripts satisfying these conditions was 134,253, comprising 107,947 protein-coding and 26,306 lncRNA transcripts. Protein-coding genes from RefSeq have an average of 6.1 annotated transcripts (maximum=113), while lncRNA loci have an average of 1.8 transcripts (maximum=32). We then used these transcripts to compute the statistics shown in **Figures S1-S5**.

We evaluated the full set of 9,795 assembled GTEx RNA-seq samples to determine a variety of metrics for the RefSeq annotations. A large majority of protein coding transcripts were detected in multiple samples: only 0.7% appeared in just one sample; 5.4% appeared in <10 samples; and all the rest appeared in 10 or more samples, with 79.1% appearing in >100 samples. Similarly, for lncRNAs, 1.9% appeared in just one sample, 10.3% appeared in <10 samples, and the rest appeared in 10 or more samples, with 50.3% appearing in >100 samples. **Figures S1** and **S2** show these results as a cumulative distribution (Fig. S1) and as a log-histogram (Fig. S2).

We next considered how many body sites each transcript appeared in. **Figure S3** shows what percentage of transcripts occurred in 1, 2, 3, etc. up to all 54 body sites. Protein-coding transcripts showed a strong tendency to appear in nearly all body sites and tissues, with >25% of these transcripts occurring in all 54 sites. In contrast, nearly 30% of lncRNAs appeared in just one body site, and pseudogenes were even more skewed, with 45% appearing in just one site.

**Figure S4** shows the distribution of expression levels for all transcripts, measured as the maximum expression level for each transcript in any site where it was observed. Expression level was measured as transcripts per million (TPM), and is shown on a log scale to accommodate outliers with very high TPM values. Protein-coding transcripts showed the highest average maximum TPM (~10), while lncRNAs were slightly lower and pseudogenes were the lowest.

To provide another view of these distributions, **Figure S5** shows them as box plots, without a log scale. From the boxplots, we can see that lncRNA outliers include transcripts above 13.87 TPM. We used this value as a (conservative) threshold to identify novel lncRNAs to include in the CHESS gene catalog.

**Multiple sequence alignments of protein coding genes.** Supplementary Figures **S6-S8** show multiple sequence alignments of a sample of novel CHESS proteins to the comprehensive protein database (**nr)** at NCBI. Initial matches were identified using BlastP ^1^, and the top animal hits were then re-aligned using muscle ^2^. Some of the non-human sequences were trimmed to remove unaligned regions on either end of the predicted proteins. The figures were created with the Seaview alignment software ^3^.

**Mass spectrometry experiments.** We searched the unmatched MS/MS spectra from a previous study ^4^ against a set of translated ORFs from all assembled transcripts (most of which did not make it into the CHESS database) that either (a) had a protein hit to a mammalian protein in GenBank or in UniProtKB/Swiss-Prot (1335 transcripts), or (b) matched transcripts that were marked as potentially coding in the FANTOM database (2115 additional transcripts). We identified peptides matching 6 of the novel CHESS proteins. Peptides corresponding to these 6 proteins were synthesized, and only 4 out of these 6 peptides could be validated upon comparison of the MS/MS spectra of experimental and synthetic peptides. **Table S6** shows the four novel ORFs confirmed by peptide evidence from proteomics data analysis, as well as two transcripts that were not validated by the MS/MS spectra. The two transcripts that were not validated are also shown in **Figure S9** and correspond to transcripts present in the FANTOM database, but excluded from the CHESS database because they did not pass our filters.

| **Table S1**. Number of exons per transcript across all assembled transcripts from all 9,795 GTEx samples. | |
| --- | --- |
| Number of exons | Number of assembled transcripts |
| 1 | 19,014,285 |
| 2 | 1,269,348 |
| 3 | 1,003,837 |
| 4 | 928,509 |
| 5 | 866,864 |
| 6 | 797,993 |
| 7 | 734,067 |
| 8 | 671,757 |
| 9 | 611,641 |
| 10-99 | 4,905 |
| 100-199 | 10,447 |
| 200+ | 3,121 |

| **Table S2**. List of RNA-seq samples used in this study, broken down by body site. | |
| --- | --- |
| Body site | Number of samples |
| Adipose-Subcutaneous | 386 |
| Adipose-Visceral (Omentum) | 235 |
| Adrenal Gland | 161 |
| Artery-Aorta | 250 |
| Artery-Coronary | 141 |
| Artery-Tibial | 363 |
| Bladder | 13 |
| Brain-Amygdala | 83 |
| Brain-Anterior cingulate cortex (BA24) | 100 |
| Brain-Caudate (basal ganglia) | 135 |
| Brain-Cerebellar Hemisphere | 120 |
| Brain-Cerebellum | 147 |
| Brain-Cortex | 133 |
| Brain-Frontal Cortex (BA9) | 122 |
| Brain-Hippocampus | 104 |
| Brain-Hypothalamus | 104 |
| Brain-Nucleus accumbens (basal ganglia) | 125 |
| Brain-Putamen (basal ganglia) | 105 |
| Brain-Spinal cord (cervical c-1) | 76 |
| Brain-Substantia nigra | 72 |
| Breast-Mammary Tissue | 222 |
| Cells-EBV-transformed lymphocytes | 139 |
| Cells-Leukemia cell line (CML) | 102 |
| Cells-Transformed fibroblasts | 306 |
| Cervix-Ectocervix | 6 |
| Cervix-Endocervix | 5 |
| Colon-Sigmoid | 175 |
| Colon-Transverse | 211 |
| Esophagus-Gastroesophageal Junction | 177 |
| Esophagus-Mucosa | 340 |
| Esophagus-Muscularis | 291 |
| Fallopian Tube | 7 |
| Heart-Atrial Appendage | 219 |
| Heart-Left Ventricle | 274 |
| Kidney-Cortex | 38 |
| Liver | 142 |
| Lung | 381 |
| Minor Salivary Gland | 71 |
| Muscle-Skeletal | 478 |
| Nerve-Tibial | 335 |
| Ovary | 112 |
| Pancreas | 203 |
| Pituitary | 127 |
| Prostate | 122 |
| Skin-Not Sun Exposed (Suprapubic) | 273 |
| Skin-Sun Exposed (Lower leg) | 401 |
| Small Intestine-Terminal Ileum | 106 |
| Spleen | 121 |
| Stomach | 211 |
| Testis | 209 |
| Thyroid | 368 |
| Uterus | 93 |
| Vagina | 99 |
| Whole Blood | 456 |

| **Table S3.** Counts for different types of genes and genomic features in RefSeq for human genome release GRCh38.p8. | | | | |
| --- | --- | --- | --- | --- |
| **Gene biotype** | **Primary Assembly** | **Alternate scaffolds and patches only** | **Mitochondrion only** | **Total count** |
| protein_coding | 19964 | 77 | 13 | 20054 |
| lncRNA | 14672 | 116 |  | 14788 |
| pseudogene | 14650 | 45 |  | 14695 |
| V_segment | 237 | 9 |  | 246 |
| J_segment | 97 |  |  | 97 |
| D_segment | 32 |  |  | 32 |
| C_region | 21 |  |  | 21 |
| rRNA | 21 |  | 2 | 23 |
| tRNA | 423 | 5 | 22 | 450 |
| snoRNA | 431 |  |  | 431 |
| snRNA | 62 |  |  | 62 |
| misc_RNA | 1208 | 6 |  | 1215 |
| antisense_RNA | 23 |  |  | 23 |
| miRNA | 1879 |  |  | 1879 |
| telomerase_RNA | 1 |  |  | 1 |
| guide_RNA | 28 |  |  | 28 |
| vault_RNA | 3 |  |  | 3 |

| **Table S4.** Number of loci in which different human genes from RefSeq and CHESS 2.0 appear, including patches and alternative loci from the GRCh38.p8 assembly. | | | | | | | |  |
| --- | --- | --- | --- | --- | --- | --- | --- | --- |
| **Gene biotype** | | | **RefSeq** | **CHESS 2.0** | | | |  |
| protein_coding | | | 22,554 | 22,930 | | | |  |
| lncRNA | | | 15,779 | 19,901 | | | |  |
| **Table S5.** Novel genes in CHESS that are over-expressed in testis and that overlap retroposons. | | | | | | | | |
| **Gene (CHESS ID)** | **Gene status** | **Chromosome** | | | **Start** | **End** | **Strand** | |
| CHS.16690 | noncoding | chr15 | | | 20201744 | 20204590 | + | |
| CHS.25209 | noncoding | chr19 | | | 1498610 | 1503544 | - | |
| CHS.2608 | noncoding | chr1 | | | 114802171 | 114804333 | - | |
| CHS.26654 | noncoding | chr19 | | | 38854720 | 38857106 | - | |
| CHS.26780 | noncoding | chr19 | | | 41179731 | 41181830 | + | |
| CHS.28763 | noncoding | chr2 | | | 10901723 | 10904345 | - | |
| CHS.31002 | noncoding | chr2 | | | 143912967 | 143922989 | - | |
| CHS.31306 | noncoding | chr2 | | | 170141600 | 170157570 | + | |
| CHS.3210 | noncoding | chr1 | | | 154287714 | 154288429 | + | |
| CHS.33245 | coding | chr20 | | | 30290598 | 30292606 | + | |
| CHS.33347 | noncoding | chr20 | | | 33693153 | 33700858 | + | |
| CHS.33383 | noncoding | chr20 | | | 35153144 | 35171346 | + | |
| CHS.33476 | coding | chr20 | | | 38456875 | 38462702 | + | |
| CHS.35376 | noncoding | chr22 | | | 23844623 | 23856404 | + | |
| CHS.35679 | noncoding | chr22 | | | 34624905 | 34633942 | - | |
| CHS.36224 | noncoding | chr22 | | | 49988882 | 49993299 | - | |
| CHS.40741 | noncoding | chr4 | | | 78548288 | 78549057 | + | |
| CHS.42538 | noncoding | chr5 | | | 32204281 | 32213665 | + | |
| CHS.42859 | noncoding | chr5 | | | 56013914 | 56015998 | - | |
| CHS.44831 | noncoding | chr5 | | | 176214805 | 176216390 | + | |
| CHS.47373 | noncoding | chr6 | | | 122847704 | 122850303 | - | |
| CHS.50862 | noncoding | chr7 | | | 73281719 | 73283865 | - | |
| CHS.51023 | noncoding | chr7 | | | 77531782 | 77536886 | - | |
| CHS.51157 | noncoding | chr7 | | | 91880804 | 91890363 | + | |
| CHS.51158 | coding | chr7 | | | 91885582 | 91889945 | - | |
| CHS.54359 | coding | chr8 | | | 123313088 | 123319527 | + | |
| CHS.55887 | noncoding | chr9 | | | 83726863 | 83738912 | - | |
| CHS.56391 | coding | chr9 | | | 107252061 | 107262261 | + | |
| CHS.56392 | noncoding | chr9 | | | 107256097 | 107258084 | - | |
| CHS.57753 | noncoding | chrX | | | 30600977 | 30607730 | + | |
| CHS.58258 | noncoding | chrX | | | 72177800 | 72179996 | + | |
| CHS.6396 | noncoding | chr10 | | | 69991463 | 69992790 | + | |
| CHS.6445 | coding | chr10 | | | 71868689 | 71876690 | + | |
| CHS.788 | noncoding | chr1 | | | 23935200 | 23939589 | + | |
| CHS.8829 | noncoding | chr11 | | | 60072557 | 60073082 | - | |
| CHS.914 | coding | chr1 | | | 26817347 | 26819141 | + | |
| CHS.9561 | noncoding | chr11 | | | 77484833 | 77491147 | - | |
| CHS.9657 | noncoding | chr11 | | | 86109431 | 86123326 | + | |
| CHS.10893 | noncoding | chr12 | | | 8624837 | 8625679 | + | |
| CHS.1134 | noncoding | chr1 | | | 33053402 | 33058967 | + | |
| CHS.12147 | noncoding | chr12 | | | 70201082 | 70203571 | + | |
| CHS.1473 | noncoding | chr1 | | | 45220909 | 45224791 | + | |
| CHS.15997 | noncoding | chr14 | | | 76896896 | 76904993 | - | |
| CHS.16077 | noncoding | chr14 | | | 82787956 | 82815139 | - | |
| CHS.17819 | noncoding | chr15 | | | 74119743 | 74126255 | + | |
| CHS.19359 | noncoding | chr16 | | | 23452749 | 23453937 | + | |
| CHS.21419 | noncoding | chr17 | | | 15860836 | 15869053 | + | |
| CHS.24071 | noncoding | chr18 | | | 6706388 | 6708835 | + | |
| CHS.24239 | noncoding | chr18 | | | 14382264 | 14386522 | - | |
| CHS.26653 | noncoding | chr19 | | | 38852074 | 38857389 | + | |
| CHS.27840 | noncoding | chr19_GL383576v1_alt | | | 73655 | 76851 | + | |
| CHS.283 | noncoding | chr1 | | | 7946565 | 7952890 | - | |
| CHS.28819 | noncoding | chr2 | | | 15678960 | 15681050 | - | |
| CHS.29059 | noncoding | chr2 | | | 27539964 | 27541202 | - | |
| CHS.31931 | noncoding | chr2 | | | 212733118 | 212772806 | + | |
| CHS.35026 | noncoding | chr21 | | | 45783658 | 45786130 | + | |
| CHS.36540 | noncoding | chr3 | | | 9559475 | 9560197 | - | |
| CHS.37291 | noncoding | chr3 | | | 49931758 | 49940035 | - | |
| CHS.38647 | noncoding | chr3 | | | 149444631 | 149448558 | - | |
| CHS.41673 | noncoding | chr4 | | | 157635854 | 157638123 | - | |
| CHS.41796 | noncoding | chr4 | | | 170045716 | 170047616 | + | |
| CHS.46507 | noncoding | chr6 | | | 47444105 | 47460321 | - | |
| CHS.48954 | noncoding | chr6_GL000253v2_alt | | | 4065766 | 4075074 | - | |
| CHS.50184 | noncoding | chr7 | | | 27044246 | 27046023 | + | |
| CHS.51980 | noncoding | chr7 | | | 139226012 | 139226471 | + | |
| CHS.56286 | noncoding | chr9 | | | 100392236 | 100394037 | - | |
| CHS.9549 | noncoding | chr11 | | | 76809730 | 76824403 | - | |

| **Table S6:** Four novel protein-coding genes (rows 1-4) along with the corresponding peptides identified by mass spectrometry. Rows 5-6 show two transcripts assembled as part of this study that did not pass all filters and were not considered protein-coding. Peptides from these transcripts were synthesized but the MS/MS spectra did not validate the identified peptide. | | | | | |
| --- | --- | --- | --- | --- | --- |
|  | **CHESS ID or FANTOM ID** | **Related sequence** | **Domains** | **Peptide sequence** | **Validated**  **by synthetic peptides?** |
| 1. | CHS.57705 | None | TM | IDISFHR | Yes |
| 2. | CHS.24083 | None | - | QLLTGAR | Yes |
| 3. | CHS.53541 | LYR motif-containing protein 4 isoform 1 (NP_001158312.1) | - | MAGLLGR | Yes |
| 4. | CHS.16287 | EAW81604.1  hCG2038093, partial | - | QLSLLGK | Yes |
| 5. | MICT00000037600.1 | Transmembrane protein 161B isoform X12 (XP_016864593.1) | - | VLGITWK | No |
| 6. | FTMT20200001077.1 | Signal peptidase complex subunit 2 (SPCS2) pseudogene | - | GFDDKYTLK | No |

**References**

1. Altschul, S.F. *et al.* Gapped BLAST and PSI-BLAST: a new generation of protein database search programs. *Nucleic Acids Res* **25**, 3389-402 (1997).

2. Edgar, R.C. MUSCLE: multiple sequence alignment with high accuracy and high throughput. *Nucleic Acids Res* **32**, 1792-7 (2004).

3. Gouy, M., Guindon, S. & Gascuel, O. SeaView version 4: A multiplatform graphical user interface for sequence alignment and phylogenetic tree building. *Mol Biol Evol* **27**, 221-4 (2010).

4. Kim, M.S. *et al.* A draft map of the human proteome. *Nature* **509**, 575-81 (2014).

5. Ota, T. *et al.* Complete sequencing and characterization of 21,243 full-length human cDNAs. *Nat Genet* **36**, 40-5 (2004).

**Figure S1**. Empirical cumulative distribution function of RefSeq transcripts, showing what percentage of transcripts (y axis) appeared in a given number of samples (x axis) or fewer. The GTEx data contain 9,795 samples in total. The left plot shows the distribution for lncRNAs, center plot shows protein-coding transcripts, and right plot shows pseudogenes.

**Figure S2**. Log-histogram of expressed transcripts in samples. The x-axis (log scale) shows the number of GTEx samples in which a transcript was observed, and the y-axis shows the number of RefSeq transcripts (out of 107,947 protein coding and 26,306 lncRNA transcripts) found in that many samples.

**Figure S3**. Histogram of expressed transcripts in the 54 different body sites. Left: frequencies of lncRNA transcripts. Center: frequencies of protein coding transcripts. Right: frequencies of pseudogene transcripts. Note the different y-axis scale for pseudogenes.

**Figure S4.** Maximum expression level (transcripts per million, or TPM) distributions for lncRNAs, protein coding genes, and pseudogenes. Shown are the percentage of transcripts (y-axis) with a range of TPM (transcripts per million) expression levels. TPM is shown on a log scale for clarity.

**Figure S5**. Boxplot distributions of maximum TPM values for transcripts from lncRNAs, protein-coding genes, and pseudogenes. Outliers are shown as dots above the plots; the protein-coding outliers fall much higher on the scale and are not shown. The lowest of the outliers for lncRNAs has a TPM of 13.87.

**
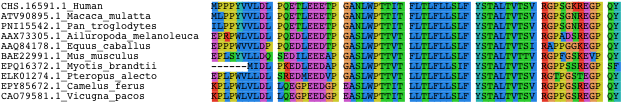
**

**Figure S6.** Multiple alignment of CHS.16591.1 (3 exons, chr 14) and genes found in rhesus macaque, chimpanzee, panda, horse, mouse, Brandt's bat, Bactrian camel, and alpaca.


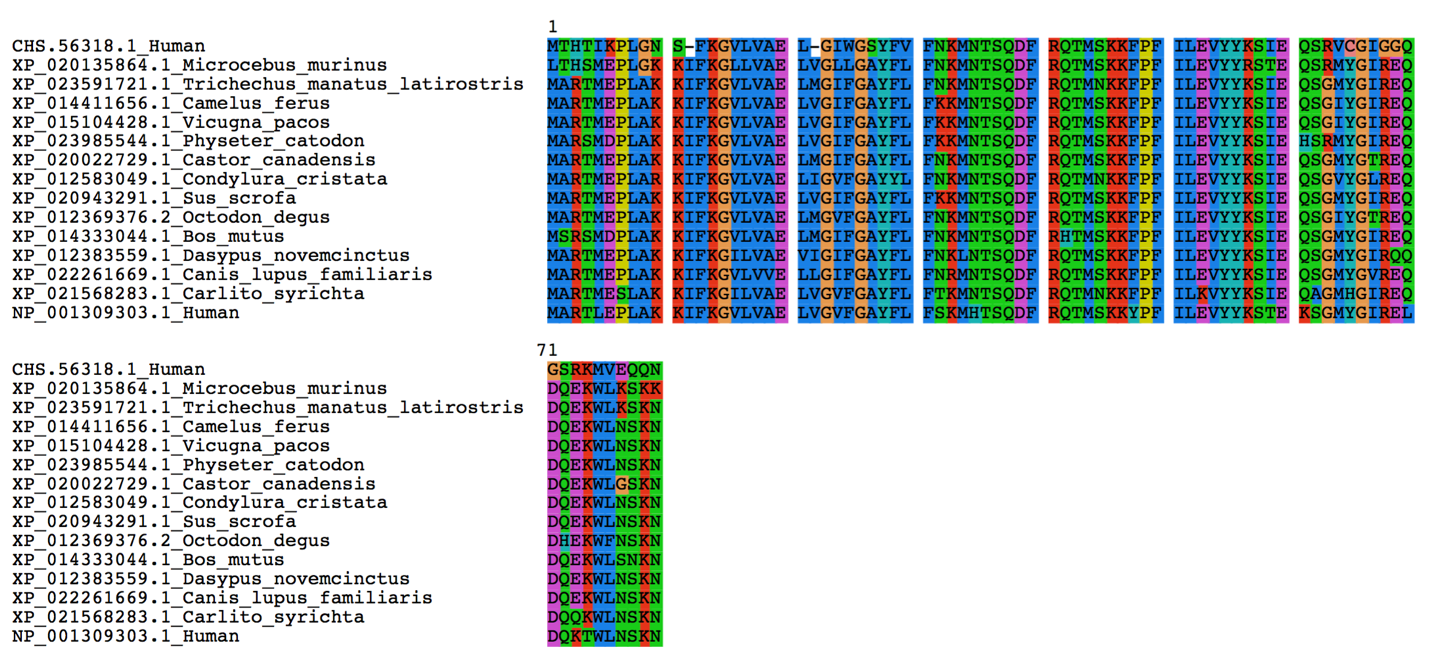


**Figure S7**. Multiple alignment of CHS.56318.1 (3 exons, chr 9) and genes found in the grey mouse lemur, sea cow, camel, alpaca, whale, beaver, mole, wild boar, mouse, wild yak, nine-banded armadillo, dog, Phillipine tarsier, and human. 36 amino acids were trimmed from the beginning of the CHESS protein. The human gene, NP_001309303, is a 6-exon gene on chr 2 annotated as CEBPZOS, CCAAT enhancer-binding protein zeta.

**
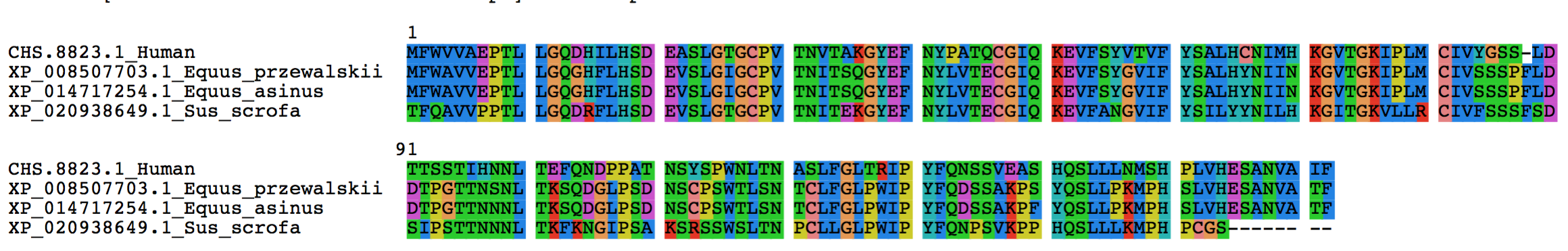
Figure S8**. Multiple alignment of CHS.8823.1 (5 exons, chr 11) and genes found in Przewalski's horse, ass, and wild boar.

**Figure S9**. Annotated MS/MS spectra of additional peptides identified for novel CHESS protein-coding genes that were confirmed (A and B) or could not be validated (C and D) by comparison of experimental spectra with those obtained from synthetic peptides.

**Figure S10.** Distributions of expression levels of transcripts in lncRNA genes (left) and protein-coding genes (right), shown for the known genes versus the novel genes in CHESS. For any transcript that appeared in more than one sample, the maximum TPM of that transcript was used to represent its expression level.

**Figure S11.** Histograms showing the number of samples (x-axis) in which transcripts from lncRNAs (left) and protein-coding genes (right) are expressed. The two upper panels show distributions for previously known transcripts, and the lower panels show distributions for transcripts unique to CHESS.

**Figure S12.** Comparison of the exons shared by and unique to CHESS (v2.1), RefSeq (rel 108), and Gencode (v28). Exons were considered shared if and only if their 5' and 3' boundaries matched precisely between a pair of databases. Note that 286,636 of the 321,618 exons (89%) that are unique to Gencode are either initial or final exons, meaning they only differed by the position of the start or termination of transcription.
